# Genetic Risk Analysis of Coronary Artery Disease in a Population-based Study in Portugal, Using a Genetic Risk Score of 31 Variants

**DOI:** 10.5935/abc.20180107

**Published:** 2018-07

**Authors:** Andreia Pereira, Maria Isabel Mendonça, Sofia Borges, Sónia Freitas, Eva Henriques, Mariana Rodrigues, Ana Isabel Freitas, Ana Célia Sousa, António Brehm, Roberto Palma dos Reis

**Affiliations:** 1 Unidade de Investigação, Hospital Dr. Nélio Mendonça, Funchal - Portugal; 2 Laboratório de Genética Humana, Universidade da Madeira, Funchal - Portugal; 3 Faculdade de Ciências Médicas, Universidade Nova de Lisboa, Lisboa - Portugal

**Keywords:** Coronary Artery Disease / history, Coronary Artery Disease / morbidity, Mortality, Polymorphism, Genetic, Epidemiology, Risk Factors

## Abstract

**Background:**

Genetic risk score can quantify individual’s predisposition to coronary artery disease; however, its usefulness as an independent risk predictor remains inconclusive.

**Objective:**

To evaluate the incremental predictive value of a genetic risk score to traditional risk factors associated with coronary disease.

**Methods:**

Thirty-three genetic variants previously associated with coronary disease were analyzed in a case-control population with 2,888 individuals. A multiplicative genetic risk score was calculated and then divided into quartiles, with the 1st quartile as the reference class. Coronary risk was determined by logistic regression analysis. Then, a second logistic regression was performed with traditional risk factors and the last quartile of the genetic risk score. Based on this model, two ROC curves were constructed with and without the genetic score and compared by the Delong test. Statistical significance was considered when p values were less than 0.05.

**Results:**

The last quartile of the multiplicative genetic risk score revealed a significant increase in coronary artery disease risk (OR = 2.588; 95% CI: 2.090-3.204; p < 0.0001). The ROC curve based on traditional risk factors estimated an AUC of 0.72, which increased to 0.74 when the genetic risk score was added, revealing a better fit of the model (p < 0.0001).

**Conclusions:**

In conclusion, a multilocus genetic risk score was associated with an increased risk for coronary disease in our population. The usual model of traditional risk factors can be improved by incorporating genetic data.

## Introduction

Coronary artery disease (CAD) has become a major health problem worldwide, with increasing prevalence and high morbidity and mortality. Traditional risk factors (TRFs) are insufficient to identify asymptomatic high-risk individuals. Epidemiology and family studies have long documented that approximately 50% of the susceptibility for heart disease is genetic.^1^ Knowledge of genetic predisposition to cardiac disease is crucial for its comprehensive prevention and treatment.

Although much of the genetic basis of coronary disease remains to be discovered, some progress has been made using both candidate gene and genome-wide association studies (GWAS).^2^ In fact, a number of genetic variants have been previously identified at several genomic regions associated with CAD.^2^

Until now, the risk attributable to any individual variant has been modest. However, discovering and combining multiple loci with modest effects into a global genetic risk score (GRS) could improve the identification of high-risk populations and improve individual risk assessment.

Therefore, the purpose of this work was to generate a multilocus GRS based on common variants previously shown to be associated with CAD, and evaluate whether it is independent of TRFs and improves the predictive ability of a model based only on TRFs.

## Methods

### Study Population

Study population was enrolled from GENEMACOR (GENEs in Madeira Island Population with CORonary artery disease), a population-based ongoing case-control registry of CAD with 2,888 participants, 1,566 cases (mean age 53.3 ± 8.0 years, 79.1% male) and 1322 controls (mean age 52.7 ± 7.8 years, 76.4% male). Cases were selected from patients discharged after being admitted for myocardial infarction/unstable angina diagnosed according to the previously described criteria,^3^ or with CAD confirmed by coronary angiography with ≥ 1 coronary lesions of ≥ 70% stenosis in ≥ 1 major coronary artery or its primary branches. Absent or non-flow limiting atheroma was excluded from the analysis. The control group consisted of healthy volunteers, without symptoms or history of CAD, selected from the same population. All controls underwent clinical assessment of conventional cardiovascular risk factors, an electrocardiogram (ECG), and, in doubtful cases, an exercise stress test, a stress echocardiography or computerized tomography for calcium scoring. Cases and controls were matched for gender and age.

Inclusion criteria comprised an age limit of 65 years and being a permanent resident to avoid genetic admixture. Principal Component Analysis (PCA)^4^ was used for analysis of population stratification for possible genetic admixture and detection of significant genetic outliers (< 5%).^4^

The study was approved by the Hospital ethics committee according to the Declaration of Helsinki and all patients provided written informed consent.

### Data collection

Data was collected from all subjects in a standardized file comprising demographic, clinical characteristics and TRFs traditional risk factors (gender, age, level of exercise, smoking status, arterial hypertension, dyslipidemia, diabetes, family history of CAD, body mass index (BMI), heart rate and pulse wave velocity (PWV).

“Smokers” referred to current smokers or subjects with less than 5 years of smoking cessation.^5^

Essential hypertension was considered when patients, at the entry into this study, were already diagnosed and/or had been on antihypertensive medication for more than 3 months or newly diagnosed hypertensives with systolic blood pressure (SBP)/diastolic blood pressure (DBP) ≥ 140/90 mmHg measured on at least 3 occasions.^6^

Dyslipidemia was defined for control population as low-density lipoprotein (LDL) > 140 mg/dL, high-density lipoprotein (HDL) < 45 mg/dL for women and < 40 mg/dL for men, Triglycerides > 150 mg/dL and apolipoprotein (Apo) B > 100 mg/dL. For patients (at high risk) dyslipidemia was considered when LDL > 100, HDL < 45 mg/dL for women and < 40 mg/dL for men, triglycerides > 150 mg/dL, Apo B > 100 mg/dL and non-HDL (total cholesterol-HDL) > 130 mg/dL.^7^

Subjects were classified as having diabetes if they were taking oral anti-diabetic medication or insulin or if their fasting plasma glucose was higher than 7.0 mmol/L or 126 mg/dL.^8^

Subjects were considered to have a family history of premature cardiovascular disease (CVD) if the father or brother had been diagnosed with CVD under the age of 55 or mother or sister under the age of 65.

The definition of other TRFs was based on the standard criteria, as previously reported.^9,10^

### Biochemical analysis

Blood samples were extracted after 12 hours’ fasting. Biochemical analyses were performed at the Central Laboratory of the Hospital, according to standard techniques. In order to determine total cholesterol, HDL, LDL, triglycerides and glucose, blood samples were placed in dry tubes, centrifuged half an hour later at 3,500 g and subsequently quantified by an enzymatic technique using an “AU 5400” (Beckman Coulter) autoanalyzer. Biochemical markers such as lipoprotein-a - Lp(a), (Apo B), and high-sensitivity C-reactive protein (hs-CRP) were quantified by immunoturbidimetry also using an “AU 5400” (Beckman Coulter) automatic system.

### Single Nucleotide Polymorphisms (SNP) selection

Two parallel approaches were employed to identify SNPs for the GRS. In the first approach, we searched the National Human Genome Research Institute database, which included SNPs identified by means of GWAS and catalogued based on phenotype and/or trait. We searched for the keywords: “coronary artery disease”, “coronary disease”, “myocardial infarction” and “early myocardial infarction.” The second approach included SNPs that were identified through candidate gene approaches, included in a published GRS for CAD.

Including criteria included genes described in previous studies with an Odds Ratio (OR) for CAD ≥ 1.1 and a minor allele frequency (MAF) > 5%. Genes with low Hardy-Weinberg equilibrium (p < 0.002) (after Bonferroni correction) were excluded.

In total, 33 SNPs were selected according to their possible CAD-related function: association with cell cycle, cellular migration and inflammation (rs1333049 (9p21.3), rs4977574 (CDKN2B), rs618675 (GJA4), rs17228212 (SMAD3), rs17465637 (MIA3), rs12190287 (TCF21), rs3825807 (ADAMTS7), rs11556924 (ZC3HC1), rs12526453 (PHACTR1)); genes involved in pro-oxidative status (rs1801133 (MTHFR 677), rs1801131 (MTHFR 1298), rs705379 (PON 1), rs662 (PON 192), rs854560 (PON 55), rs6922269 (MTHFD1L); genes associated with modifiable risk factors such as lipids metabolism, hypertension and diabetes/obesity (rs3798220 (LPA), rs2114580 (PCSK9), rs20455 (KIF6), rs7412/rs429358 (APOE), rs964184 (ZNF259), rs599839 (PSRC1), rs5186 (AT1R), rs699 (AGT), rs4340 (ACE), rs4402960 (IGF2BP2), rs1326634 (SLC30A8), rs266729 (ADIPOQ), rs7903146 (TCF7L2), rs17782313 (MC4R), rs1801282 (PPARG), rs1884613 (HNF4A), rs8050136 (FTO) and rs1376251 (TAS2R 50)) (Supplementary Table 1).

### Genetic analyses

Genetic analyses were performed at the Human Genetics Lab of the University of Madeira. Genomic DNA was extracted from 80 µl of peripheral blood using a standard phenol-chloroform method. A TaqMan allelic discrimination assay for genotyping was performed using labelled probes and primers pre-established by the supplier (TaqMan SNP Genotyping Assays, Applied Biosystems).

All reactions were done on an Applied Biosystems 7300 Real Time PCR System and genotypes were determined using the 7300 System SDS Software (Applied Biosystems, Foster City, USA) without any prior knowledge of individual’s clinical data. Quality of genotyping techniques was controlled by the inclusion of one non-template control (NTC) in each plate of 96 wells. All SNPs TaqMan assays had blind duplicates accounting for 20% of all samples. Some SNP genotypes were randomly confirmed by conventional direct DNA sequencing, as 10-15% of all samples were re-amplified for sequencing. Call rates for SNPs in the GRS were 98%-100% and a minimum 95% call rate was set for quality control.

### Computation of the GENETIC RISK SCORE

We have tested several models to construct the GRS using both non-weighted and weighted scores, taking into consideration each pattern of inheritance for each gene locus. An additive score (AGRS) was generated, i.e., for each one of the 31 variants a score of 0, 1, and 2 was defined as there were 0, 1 or 2 risk alleles, by calculating the accumulated sum of the risk alleles in these variants. Each individual could be assigned a GRS of 0-62. Additionally, a multiplicative GRS (MGRS) was calculated by multiplying the relative risk for each genotype.

Validation of the risk score calculation was performed in a random sample of 597 patients (20%).

### Statistical analysis

Categorical variables were expressed by frequencies and percentages and compared by the Chi-squared test or Fisher’s exact test. Continuous variables were expressed as mean ± standard deviation (SD) or median (1st quartile - 3rd quartile) and compared by Student's t-test (unpaired) or Mann-Whitney, as appropriate. The Kolmogorov-Smirnov test and the Levene´s test were used to test the assumption of normality and the homogeneity of the variables. All analyses were considered significant when p values were less than 0.05.

Binary logistic regression was used to determine the combined and separate effects of the variables on the risk for angiographic CAD. GRS was modeled using as a continuous variable and as quartiles, using the first quartile as the reference category. Multivariate analyses were used to adjust for 7 covariates also reported to be associated with CAD. We plotted receiver operating characteristic (ROC) curves and calculated the area under the curve (AUC) for logistic regression models including TRFs without and with GRS (quartiles). Pairwise comparison of ROC curves was performed using the Delong test.^11^ The model calibration was tested with Hosmer-Lemeshow goodness-of-fit test. A P-value less than 0.05 was considered statistically significant. Collinearity between the variables was measured by assessment of tolerance and variance inflation factor (VIF).

Associations of SNPs with CAD were considered significant at p < 0.05 and in aggregate with GRS models at p < 0.0015 applying Bonferroni correction.

For MAF of 30%, the study had 70% power to detect an OR for CAD of 1.3 and > 90% for OR ≥ 1.35, for 2-sided alpha of < 0.05 for 2,000 cases and 1,000 controls. Power calculations used *G power Statistical Power Analyses.*

The potential of GRS to improve individual risk stratification then was measured using the net reclassification improvement (NRI) method,^12^ defined as the percentage of subjects in each subgroup changing categories when the new model of GRS (in quartiles) was added. The integrated discrimination improvement (IDI), defined as the incremental improvement prognostic value of GRS, was compared between cases and controls. NRI was computed by categorical and non-categorical (continuous) variables using the PredictABEL package available in R software (version 3.2.0).

Statistical analyses were performed using SPSS version 19.0 (IBM), MedCalc version 13.3.3.0 and R software version 3.1.2.

## Results

### Baseline characteristics of the population

Table 1 shows the baseline characteristics of our population. As expected, cases and controls showed no significant differences concerning gender and age, since this was a selection criterion. Higher frequency of dyslipidemia, diabetes, hypertension, physical inactivity, smoking habit, alcohol consumption, and family history of premature cardiovascular disease was found in CAD patients when compared to the controls (p < 0.0001). Also, PWV, BMI and waist-to-height ratio were higher in cases than in controls, with statistical significance (p < 0.05) (Table 1). The other biochemical variables analyzed such as hemoglobin, leucocytes, fibrinogen, homocysteine and hs-CRP > 3 showed significantly higher levels in the coronary patients group when compared to the controls (p < 0.05) (Table 1).

**Table 1:** Baseline characteristics of our study population

Variables	Cases (n = 1566)	Controls (n = 1322)	P value
Age, years	53.3 ± 8.0	52.7 ± 7.8	0.053
Male Gender, n (%)	1238 (79.1%)	1010 (76.4%)	0.087
Dyslipidemia^†^, n (%)	1398 (89.3)	1103 (83.4)	0.0001
Total Cholesterol, mg/dl	180.0 (154.0 – 213.0)	205.0 (181.0 – 234.0)	< 0.0001
LDL, mg/dl	104.6 (82.8 – 128.7)	127.2 (104.7 – 152.3)	< 0.0001
HDL, mg/dl	41.0 (35.0 – 49.0)	48.0 (41.0 – 57.0)	< 0.0001
Triglycerides, mg/dl	141.0 (102.0 – 210.0)	121.0 (89.0 – 174.0)	< 0.0001
Apolipoprotein B, mg/dl	93.9 (75.5 – 113.3)	92.5 (43.0 – 115.8)	< 0.0001
Lipoprotein (a), mg/dl	20.4 (9.2 – 62.0)	12.8 (8.8 – 29.3)	< 0.0001
Diabetes, n (%)	533 (34.0)	175 (13.2)	< 0.0001
Fasting glucose, mg/dl	106.0 (96.0 – 129.0)	99.0 (91.0 – 109.0)	< 0.0001
Hypertension, n (%)	1114 (71.1)	700 (53.0)	< 0.0001
SBP, mmHg	137.9 ± 20.8	136.2 ± 18.1	0.024
DBP, mmHg	82.6 ± 11.8	83.9 ± 11.1	0.002
Heart rate, bpm	68.8 ± 12.5	72.3 ± 11.5	< 0.0001
PWV, m/s	8.6 ± 1.9	8.3 ± 1.7	< 0.0001
Smoking status^•^, n (%)	730 (46.6)	309 (23.4)	< 0.0001
Level of exercise^*^, n (%)	573 (36.6)	761 (57.6)	< 0.0001
Alcohol, g/day	24.7 ± 49.7	18.2 ± 28.2	< 0.0001
BMI, kg/m^2^	28.6 ± 4.2	28.1 ± 4.5	0.007
Waist/Height	0.61 ± 0.06	0.59 ± 0.07	< 0.0001
Family history, n (%)	373 (23.8)	167 (12.6)	< 0.0001
Hemoglobin, g/dl	14.6 (13.8 – 15.4)	14.7 (14 – 15.4)	0.001
Leucocytes, 103/µl	7.1 (6 – 8.3)	6.6 (5.6 – 7.8)	< 0.0001
Fibrinogen, mg/dl	387 (337 – 444)	361 (315 – 409)	< 0.0001
Homocysteine, µmol/L	12.2 (10 – 14.9)	11.4 (9.7 – 13.6)	< 0.0001
Hs-CRP, mg/L > 3, n (%)	648 (41.4)	496 (37.5)	0.035

† Controls: LDL > 140 mg/dL, HDL < 40 mg/dL for men and < 45 mg/dLfor women; triglycerides > 150mg/dL, APO B > 100 mg/dL. Cases: LDL > 100 mg/dL; triglycerides > 150 mg/dL, HDL < 40 mg/dL for men and < 45 mg/dL for women; APO B > 100 mg/dL, non HDL > 130 mg/dL;

* More than 40 min/week;

• Current smokers or < 5 years of cessation; HDL: high density lipoprotein; LDL: low density lipoprotein; SBP: systolic blood pressure; DBP: diastolic blood pressure; PWV: pulse wave velocity; BMI: body mass index; Hs-CRP: high sensitivity C-reactive protein. Categorical variables compared by the Chi-square test. Continuous variables expressed as mean ± standard deviation (using Student’s t-test) and biochemical variables as median (1st quartile – 3rd quartile) (using Mann-Whitney’s test). Statistical significance: p < 0.05.

### Computation and analysis of Genetic Risk Score

Deviation from Hardy-Weinberg equilibrium for the 33 genotypes at individual loci were assessed using the Chi-squared test and p < 0.002 with Bonferroni correction for all SNPs included. LPA gene variant was excluded for further analyses due to its low Hardy-Weinberg p-value (p < 0.002). Linkage disequilibrium for the mutually adjusted SNPs within the genes was studied. CDKN2B gene was excluded because of the strong linkage disequilibrium with another selected SNP, rs1333049, which resides in the 9p21 region. The remaining 31 SNPs were included for further analysis (Supplementary Table 1).

In this study, the MGRS had the highest AUC value for assessing the risk for CAD disease with a specificity of 62.3% and sensitivity of 54% (data not shown) and therefore this model was computed in the subsequent analyzes (Supplementary Table 2).

The MGRS of 31 SNPs was significantly higher in CAD cases than in controls (0.67 ± 0.73 vs 0.48 ± 0.53; p < 0.0001), even by quartile and gender discrimination (Table 2).

**Table 2:** Distribution of multiplicative genetic risk score (MGRS) for cases and controls by quartiles and gender

Variables	Cases (n = 1566)	Controls (n = 1322)	p value
MGRS	0.67 ± 0.73	0.48 ± 0.53	< 0.0001
1^st^ Quartile	0.18 ± 0.05	0.17 ± 0.05	< 0.0001
2^nd^ Quartile	0.33 ± 0.05	0.33 ± 0.05	
3^th^ Quartile	0.52 ± 0.07	0.52 ± 0.07	
4^ th^ Quartile	1.35 ± 1.02	1.18 ± 0.88	
MGRS male	0.67 ± 0.77	0.48 ± 0.44	< 0.0001
MGRS female	0.65 ± 0.58	0.51 ± 0.74	0.006

MGRS was expressed as mean ± standard deviation (SD) (using Student’s t-test). Statistical significance: p < 0.05.

**Figure 1:**
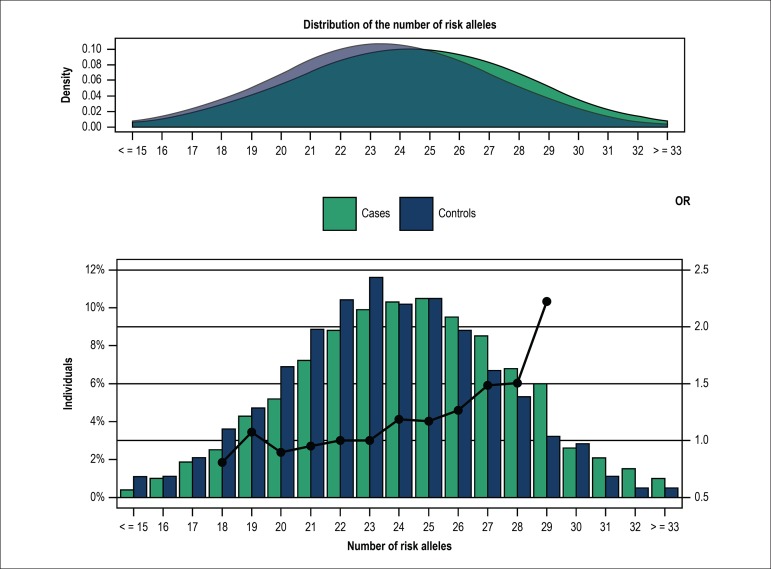
Distribution of the number of risk alleles by cases and controls. A logistic regression model was used to determine the coronary artery disease risk by the number of risk alleles compared to the number of reference alleles (23 alleles, in relation to the median value of the controls). Dots: regression analysis odds ratio for coronary artery disease.

A normal distribution of risk alleles in the total sample set including cases and controls is shown in Figure 1. While CAD patients exhibited lower GRS values, risk alleles were more prevalent in this group than in controls. In CAD patients, a mean of 27 risk alleles was seen in 52% of the individuals, and a mean of 26 risk alleles was found in 53% of controls (Figure 1).

**Figure 2:**
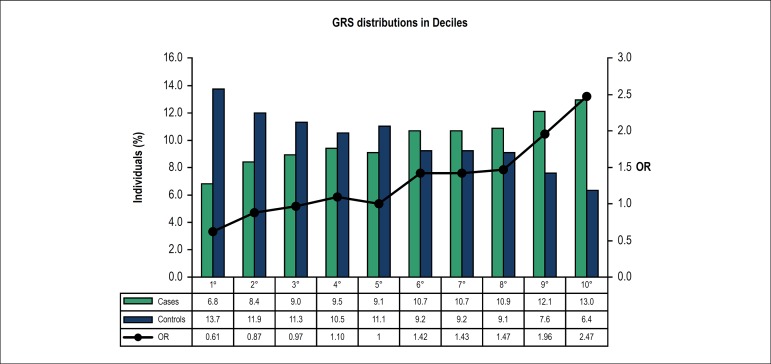
Distribution of genetic risk score in deciles by cases and controls. A logistic regression model was used with the 5th decile of the controls as the reference class.

When analyzed in deciles, GRS showed that the increase in the number of risk alleles was significantly associated with CAD as shown by inter-deciles p values (1st decile: OR = 0.612 (0.439 - 0.853), p = 0.004; 9th decile: OR =0.957 (01.400 - 2.734), p < 0.0001 and last decile: OR = 2.472 (1.755 - 3.482), p < 0.0001) (Figure 2).

A logistic regression analysis was performed with GRS quartiles, using the first as the reference category. Results showed an increase in CAD risk with statistical significance across the 2^nd^, 3^rd^ and 4^th^ quartiles with respective ORs and CIs of 1.372 (1.114 - 1.689), 1.878 (1.522 - 2.317) and 2.588 (2.090 - 3.204), respectively (data not shown).

A multivariable predictive model for CAD incorporating GRS quartiles and TRFs is presented in Table 3. The 4^th^ GRS quartile has intermediate contribution to CAD phenotype - OR = 2.727 (2.162 - 3.439), greater than dyslipidemia - OR = 1.298 (1.023 - 1.646) and hypertension - OR = 2.067 (1.744 - 2.450). The reduced contribution of dyslipidemia on CAD risk may be due to standard use of statins in CAD patients. Extended adjustment for cofounding variables (gender, age, heart rate, PWV, low exercise level, BMI and family history of CAD) revealed modest increases in the OR for TRFs and the 2^nd^ and 3^rd^ quartiles of GRS.

**Table 3:** Multivariate analysis performed with the multiplicative genetic risk score (MGRS) (quartiles) and traditional risk factors

Variables	OR* (95% CI)	p value	OR^+^ (95% CI)	p value
MGRS (Quartiles)	------	------	------	< 0.0001
2^nd^	1.355 (1.082 – 1.698)	0.008	1.406 (1.107 – 1.786)	0.005
3^rd^	1.934 (1.539 – 2.429)	< 0.0001	2.006 (1.575 – 2.554)	< 0.0001
4^th^	2.727 (2.162 – 3.439)	< 0.0001	2.657 (2.083 – 3.389)	< 0.0001
Smoking	3.440 (2.887 – 4.100)	< 0.0001	3.651 (3.030 – 4.401)	< 0.0001
Diabetes	3.138 (2.559 – 3.847)	< 0.0001	3.436 (2.763 – 4.273)	< 0.0001
Hypertension	2.067 (1.744 – 2.450)	< 0.0001	2.187 (1.816 – 2.633)	<0.0001
Dyslipidemia	1.298 (1.023 – 1.646)	0.032	1.344 (1.044 – 1.731)	0.022
Constant	0.186	< 0.0001		

Using forward Wald method (SPSS vs. 19.0); Dyslipidemia. Controls: LDL > 140 mg/dL, HDL < 40 mg/dL for men and < 45 mg/dLfor women; triglycerides> 150 mg/dL, APO B > 100 mg/dL. Cases: LDL > 100 mg/dL; triglycerides > 150 mg/dL, HDL < 40 mg/dL for men and < 45 mg/dL for women; APO B > 100 mg/dL, non HDL > 130 mg/dL;

* OR: odds ratio adjusted for age and gender;

+ OR: odds ratio adjusted for gender, age, heart rate, pulse wave velocity, sedentary life style, alcohol, body mass index and family history; CI: confidence interval; Statistically significant for p < 0.05.

We used VIF to test for multi-collinearity among the variables included in our GRS adjusted logistic regression model. Tolerance and VIF were respectively > 0.1 and < 10 attesting for no significant collinearity between variables included in the adjustment model.

**Figure 3:**
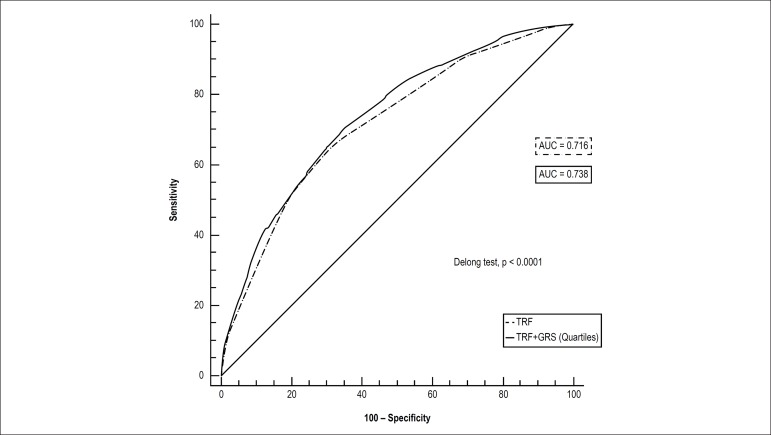
ROC curves based on the baseline model (traditional risk factors, TRFs) and after adding the genetic risk score (GRS) (quartiles) in predicting the risk for coronary artery disease. The two curves are based on logistic regression models incorporating conventional risk factors (diabetes, dyslipidemia, smoking and hypertension) with and without the GRS. AUC indicates area under curve. The Delong test compares the difference between the two AUCs (p < 0.0001).

Two ROC curves were plotted based on the TRFs without and with the GRS (Figure 3). The first ROC curve estimated an AUC of 0.72, which increased to 0.74 when the GRS was added, revealing a better fit of the model (p < 0.0001) (Figure 3).

The NRI and its *p* value were used to make conclusions about improvements in prediction performance gained by adding a set of biomarkers to an existing risk prediction model. The addition of GRS quartiles to TRF improved the risk classification of the models (Table 4). This new marker provided a continuous NRI of 31% (95% CI: 23.8-38.3%; p < 0.0001) with 14.6% reclassification of CAD patients and 16.4% of healthy control population (Table 4).

**Table 4:** The category-free net reclassification index (cfNRI) after addition of the GRS quartiles

Group	n	Higher risk n (%)	Lower risk n (%)	p (cfNRI)	cfNRI (%)	cfNRI (95% CI)
CAD patients	1566	897 (57.3%)	669 (42.7%)	< 0.0001	14.6%	(9.7-19.5%)
Healthy controls	1322	553 (41.8%)	769 (58.2%)	< 0.0001	16.4%	(11.2-21.8%)
Total	2888	---	---	< 0.0001	31%	(23.8-38.3%)

GRS: genetic Risk Score; CAD: coronary Artery Disease; CI: confidence Interval; cfNRI: category-free net reclassification index. This analysis uses the function “improveProb” from R software package “Hmisc”.

NRI was also computed using categorical variables and applied to this case-control study and was defined as the percentage of subjects changing categories in each subgroup when adding the new marker (CAD quartile score). Movement towards a better category (higher in patients than in controls) was calculated to address a potential impact for clinical use. NRI showed higher improvement capacity in reclassifying 19.5% of patients from the 50-75% category to the highest risk (75-100%) category. Likewise, 14.1% of healthy controls were moved down into a lower risk category, from 25-50% risk category to < 25% one (Table 5).

**Table 5:** Reclassification table comparing predicted coronary artery disease (CAD) risk with and without genetic risk score (GRS) quartiles

Predicted risk (without GRS)	Reclassified predicted risk (with GRS)				% Increase	%/ Decrease
CAD patients (n = 1,566)	< 25%	25-50%	50-75%	75-100%		
< 25%	6	11	0	0	0,7%	0%
25-50%	44	335	123	0	7,9%	2.8%
50-75%	0	59	471	305	19,5%	3.8%
75-100%	0	0	9	203	0%	0.6%
NRI CAD patients	20.9%					
**Healthy controls (n = 1,322)**						
< 25%	65	36	0	0	2,7%	0%
25-50%	186	504	88	0	6,7%	14.1%
50-75%	0	60	268	79	6%	4.5%
75-100%	0	0	1	35	0%	0.1%
NRI controls	3.3%					
NRI total	24.2%					

NRI: net reclassification improvement (categorical NRI); CAD: coronary artery disease.

Furthermore, the inclusion of GRS quartiles to TRF also provided an IDI of 2.5% (95%CI: 1.9-3.1%; p < 0.0001) (data not shown).

## Discussion

Several years ago, polymorphisms involved in specific biological pathways, relevant to coronary atherosclerosis, were genotyped to determine their association with CAD. This candidate gene approach revealed about 30 high-confidence SNPs loci with significant effects on atherosclerosis.^13^ However, following traditional candidate gene approach has generated many conflicting results or with weak associations; replication studies are necessary for consistent validation of these results.

In 2004, Mendonça et al. first genotyped a*ngiotensin*-converting enzyme (ACE) I/D polymorphisms in a Portuguese population yielding similar reports as described in literature.^14^

After the development of high capacity arrays in 2008,^15^ GWAS examined millions of polymorphisms simultaneously in several ethnical subpopulations with a case-control design. The standardized minimum significance level set at 1x10^-5^ added reliability to cardiovascular genetics and put it into perspective.^16^

In 2007, Samani et al.^17^ first identified chromosomal loci that were strongly associated with CAD in the Wellcome Trust Case Control Consortium (WTCCC) study (which involved 1,926 case subjects with CAD and 2938 controls) and looked for replication in the German MI (Myocardial Infarction) Family Study.^17^

In the following years, a surprisingly large number of gene variants were consistently reported to be associated with CAD. The 9p21 variant was the most frequently gene variant reported across populations. The huge consortium of Wellcome Trust and three other European research groups joined for the CARDIOGRAM project that confirmed, in a very large sample (> 22,000 cases) of individuals of European ancestry, a 29% increase in risk for MI per copy of the rs1333049 9p21 variant (p = 2×10^-20^).^18^

Our research group replicated this 9p21 variant analysis in the Portuguese population and found a CC genotype prevalence of 35.7% in CAD patients, with an adjusted OR of 1.34, p = 0.010. The adjusted OR for TRF of CC genotype was 1.7 (p = 0.018) and CG genotype of OR = 1.5, p = 0.048. The authors concluded that although the mechanism underlying the risk is still unknown, the robustness of this risk allele in risk stratification for CAD has been consistent, even in very different populations. The presence of the CC or CG genotype may thus prove to be useful for predicting the risk of developing CAD in the Portuguese population.^19^

The most recent meta-analysis of GWAS for (CAD) identified 46 genome-wide loci with significant association and 104 genome-wide loci potentially associated with increased risk.^20,21^

In our study, we found a gradual and continual increase in CAD risk with increasing number of CAD risk alleles carried. Individuals in the bottom decile are naturally protected and subjects in top decile of the GRS had a CAD risk of 2.472 (1.755 - 3.482). Even though the score distribution overlaps between cases and controls, the GRS is significantly associated with CAD risk and can be used to identify subjects at highest risk in terms of lifestyle or therapeutic interventions.

Our results are similar to others reports in Caucasians populations where GRS with 13, 29 or 109 SNPs^22-24^ were independent and marginally increased the predictive power of TRF conferred either by AUC increases, C-index changes or more modern discriminative statistical methods like reclassification measures or improved discrimination.

We report a higher OR for the 4^th^ quartile of GRS (2.59) compared to 1.66 reported by Ripatti et al. in the highest quintile.^22^ When comparing the relative weight of the GRS in the multivariate logistic analysis we found slightly lower OR than smoking, hypertension, and dyslipidemia. In Ripatti´s^22^ cohort, a weighted GRS was also an independent predictor even after adjusting for age, sex and TRFs in a Northern European population-based trial. The relative risk of the GRS based on 13-SNP was also lower than that of dyslipidemia and comparable to the effects of hypertension.^22^

An increased power to TRFs definition has been given in this study. For instance, we have used a broad dyslipidemia term including Apo B levels as indicated by 2016 lipid guidelines.^7^ Moreover, we have not considered ex-smokers until 5 years of cessation to account for the risk for CV disease events decrease be comparable to a nonsmoker.^5^

Thanassoulis et al.^24^ demonstrated that adding to a 13 SNP-based GRS, 89 SNPs associated with modifiable risk factors did not increase the power of the GRS reporting a HR of 1.01 (95% CI 0.99 -1.03; p = 0.48). This revealed that the weak association of polymorphisms with CAD risk factors in GRS analysis could be masked by the relative stronger effect of other polymorphisms. Considering the lack of a significant association of lipid profiles with CAD risk, Jansen et al. reported in 2015 that several SNPs associated with type 2 diabetes mellitus were related with CAD risk.^25^ Recently, Webb et al. identified 6 new loci associated with CAD at genome-wide significance. The study confirmed a pleiotropy between lipid traits, blood pressure phenotypes, body mass index, diabetes, and smoking behavior.^26^ Our GRS is an assembly of risk factors and non-risk factors-related SNP, reinforcing the genotype-phenotype interactions.

### Limitations of this study

The main clinical utility of the GRS in our population is a modest improvement in risk stratification. GRS seems to be a better indicator of patients at a higher than average risk for DAC as compared with TRF stratification. The number and type of SNPs included is limited in our study and a larger number of GWAS hit SNPs should be included in further studies. Nevertheless, the increasing capability of analyzing multiple SNPs in GRS so far have not been translated into increasing ability of risk prediction.

Finally, this study did not include a gene-gene (G-G) and gene-environment (G-E) analysis. It is expected that, as better statistical significance arises from those interplays, the G-G and G-E incorporation in GRS plus TRF will increase our ability to accurately and individually predict risk.

## Conclusions

 We conclude that a multilocus GRS based on multiple variants of genetic risk was associated to an increased cardiovascular risk in a Portuguese population. We found that a GRS calculated with the 31 studied SNPs was significantly associated to CAD and that 25% of individuals who carry the greatest risk alleles have, approximately, 2.5 times increased CAD risk when compared to those in the lowest quartile. This GRS has provided a slight improvement of the predictive ability compared to the initial model and can enhance individual risk stratification. These results highlight the potential value of including genetic information in the usual models.

## Appendix

**Supplementary Table 1:** List of the 33 genetic variants previously associated with coronary artery disease risk, used for the development of the genetic risk score in the study population

SNP ID	Nearest gene	Chr	Position	Genotypic OR (95%CI)	p value	Allelic OR (95%CI)	p value	MAF (%)	Potential Mechanismof Action
rs1333049	9p21.3	9	22125504	1.147 (1.036-1.270)^+^	0.008	1.155 (1.041-1.282)	0.007	45.8	Cellular
rs4977574	CDKN2B	9	22098575	1.161 (1.049-1.286)^+^	0.004	1.172 (1.056-1.302)	0.003	42.0	Cellular
rs618675	GJA4	1	34922761	1.143 (0.792-1.649)*	0.475	1.046 (0.918-1.191)	0.502	19.6	Cellular
rs17228212	SMAD3	15	65245693	1.202 (0.888-1.629)*	0.234	1.025 (0.910-1.155)	0.684	25.3	Cellular
rs17465637	MIA3	1	222650187	1.088 (0.971-1.220)^+^	0.148	1.088 (0.971-1.220)	0.147	28.6	Cellular
rs12190287	TCF21	6	134256218	1.230 (1.100-1.375)^+^	< 0.0001	1.226 (1.098-1.368)	0.0003	32.7	Cellular
rs3825807	ADAMTS7	15	76876166	1.073- (0.967-1.191)^+^	0.185	1.074 (0.967-1.194)	0.181	41.2	Cellular
rs11556924	ZC3HC1	7	130023656	1.227 (1.058-1.423)*	0.007	1.157 (1.037-1.290)	0.009	34.3	Cellular
rs1332844	PHACTR1	6	12927312	1.113 (1.003-1.235)^+^	0.044	1.113 (1.003-1.236)	0.043	44.3	Cellular
rs2114580	PCSK9	1	55167236	1.079 (0.821-1.417)*	0.587	0.974 (0.866-1.096)	0.665	26.3	Lipids
rs3798220	LPA	6	160540105	1.484 (1.212-1.816)^+^	< 0.0001	2.167 (1.452-3.235)	< 0.0001	2.1	Lipids
rs20455	KIF6	6	39357302	1.129 (0.896-1.424)*	0.306	1.060 (0.949-1.184)	0.302	32.8	Lipids
rs7412/ rs429358^1^	APOE^1^	19	44908822/44908684	1.261 (1.062-1.497)^#^	0.008	1.231 (1.056-1.435)	0.008	13.4	Lipids
rs964184	ZNF259	11	116778201	1.131 (0.986-1.298)^+^	0.078	1.130 (0.986-1.295)	0.079	17.7	Lipids
rs599839	PSRC1	1	109279544	1.059 (0.933-1.203)^+^	0.375	1.058 (0.933-1.201)	0.379	21.4	Lipids
rs1801133	MTHFR 677	1	11796321	1.178 (1.017-1.365)^#^	0.029	1.114 (0.998-1.243)	0.055	33.5	Oxidation
rs1801131	MTHFR 1298	1	11794419	0.944 (0.816-1.093)^#^	0.443	0.958 (0.854-1.075)	0.465	28.0	Oxidation
rs705379	PON -108	7	96324583	1.135 (0.950-1.355)^#^	0.163	1.068 (0.962-1.184)	0.217	46.4	Oxidation
rs662	PON 192	7	95308134	0.836 (0.652-1.072)*	0.157	0.927 (0.828-1.037)	0.186	30.1	Oxidation
rs854560	PON 55	7	95316772	1.161 (1.044-1.290)^+^	0.006	1.161 (1.044-1.290)	0.006	40.4	Oxidation
rs6922269	MTHFD1L	6	150931849	1.067 (0.804-1.416)*	0.653	0.996 (0.887-1.118)	0.943	27.3	Oxidation
rs5186	AT1R	3	148742201	1.245 (0.906-1.710)*	0.177	1.062 (0.942-1.198)	0.323	24.7	RAS
rs699	AGT	1	230710048	0.932 (0.798-1.090)^#^	0.380	0.969 (0.873-1.076)	0.552	42.9	RAS
rs4340	ACE	17	61565892	1.165 (1.001-1.355)*	0.048	1.083 (0.973-1.205)	0.143	38.1	RAS
rs4402960	IGF2BP2	3	185793899	1.124 (0.876-1.443)*	0.358	1.020 (0.911-1.141)	0.736	30.8	Diab/Obes
rs1326634	SLC30A8	8	117172544	1.213 (0.914-1.609)^#^	0.181	1.081 (0.961-1.217)	0.195	25.8	Diab/Obes
rs266729	ADIPOQ	3	186841685	1.209 (1.041-1.403)^#^	0.013	1.165 (1.030-1.318)	0.015	23.3	Diab/Obes
rs7903146	TCF7L2	10	112998590	0.961 (0.862-1.072)^+^	0.480	0.962 (0.863-1.072)	0.482	35.3	Diab/Obes
rs17782313	MC4R	18	60183864	1.314 (0.931-1.855)*	0.120	1.016 (0.896-1.152)	0.806	21.6	Diab/Obes
rs1801282	PPARG	3	12351626	1.427 (0.717-2.843)^#^	0.309	1.164 (0.970-1.396)	0.102	8.8	Diab/Obes
rs1884613	HNF4A	20	44351775	1.159 (0.987-1.360)^#^	0.072	1.106 (0.960-1.273)	0.163	16.2	Diab/Obes
rs8050136	FTO	16	53782363	1.194 (1.026-1.390)^#^	0.022	1.129 (1.016-1.255)	0.025	39.7	Diab/Obes
rs1376251	TAS2R 50	12	11030119	1.556 (0.767-3.155)*	0.217	1.080 (0.920-1.267)	0.349	11.9	Diab/Obes

SNP: Single Nucleotide Polymorphism; Chr: Chromosome; OR: Odds Ratio; CI: Confidence Interval; MAF: Minor Allele Frequency; RAS: Renin-Angiotensin System; Diab/Obes: Diabetes/Obesity;

+ Additive model;

* Recessive model;

# Dominant model;

1 Resulting from a Haplotype; Table shows susceptibility loci for coronary artery disease, genotypic and allelic ORs and p values for the lead SNP within each locus reported in genome-wide association studies and candidate gene studies. Genotypic ORs are given for additive, recessive and dominant models. Potential mechanism of action is on the basis of what is already known about the function of the nearby genes. It includes “Cellular” (genes associated to cell cycle, cellular migration and inflammation); “Oxidation” (genes involved in pro-oxidative status) and associated with modifiable risk factors such as “Lipids” metabolism, hypertension (“RAS”) and Diabetes/Obesity.

**Supplementary Table 2:** Logistic regression with respective ORs and ROC curves with respective AUCs of the GRS models

GRS models	OR (95% CI)	P value^1^	AUC (95% CI)	Sensitivity (%)	Specificity (%)	P value^2^
Multiplicative	1.78 (1.52 – 2.10)	< 0.0001	0.61 (0.59 – 0.62)	54.0	62.3	< 0.0001
Additive	1.06 (1.04 – 1.09)	< 0.0001	0.56 (0.54 – 0.58)	58.7	50.5	< 0.0001
Weighted (Best model OR)	1.02 (0.94 – 1.10)	0.660	0.57 (0.55 – 0.59)	41.0	70.3	< 0.0001
Weighted (Beta)	2.23 (1.88 – 2.65)	< 0.0001	0.60 (0.58 – 0.61)	43.0	71.5	< 0.0001
Weighted (Literature OR)	1.35 (1.12 – 1.62)	0.001	0.54 (0.52 – 0.55)	53.4	54.1	0.008
Classic weighted	3.01 (2.32 – 3.89)	< 0.0001	0.59 (0.57 – 0.61)	59.4	54.4	< 0.0001

OR: Odds ratio; ROC: Receiver Operating Characteristic; AUC: Area under curve; GRS: Genetic Risk Score; CI: Confidence interval;

1 P value: Obtained by logistic regression to evaluate the significance of the odds ratio;

2 P value: Obtained by the ROC Curve to verify the significance of the area under the curve; Statistically significant for p < 0.05.
